# Seroprevalence of *Toxoplasma gondii* and *Neospora* spp. in horse population of Tehran, Iran

**DOI:** 10.1038/s41598-024-61999-z

**Published:** 2024-07-24

**Authors:** Farzane Shams, Mohammad Jokar, Arman Abdous, Pardis Mohammadi, Aryan Abbassioun, Torsten Seuberlich, Vahid Rahmanian

**Affiliations:** 1 Graduate School for Cellular and Biomedical Sciences, Vetsuisse, University of Bern, Bern, Switzerland; 2 Faculty of Veterinary Medicine, Karaj Branch, Islamic Azad University, Karaj, Iran; 3 Department of Virology, Faculty of Veterinary Medicine, Tehran University, Tehran, Iran; 4 Department of Public Health, Torbat Jam Faculty of Medical Sciences, Torbat Jam, Iran; 5 Division of Neurological Sciences, Vetsuisse Faculty, University of Bern, Bern, Switzerland

**Keywords:** *Toxoplasma gondii*, *Neospora*, Horse, Serology, Tehran, Parasitology, Risk factors

## Abstract

*Neospora* spp. and *Toxoplasma gondii* are two closely related protozoan parasites that are widely distributed throughout the world. Horses can act as intermediate hosts for both parasites and can acquire disease. Blood samples were taken from 487 clinically healthy horses from 17 different mechanized stables in Tehran, the capital of Iran, during September and November of 2022. IFAT and ELISA were employed to detect antibodies directed against *Neospora* spp. and *T. gondii*. The anti-N. caninum antibodies were detected in 52 of the horses (10.67%) based on IFAT and in 86 of the 487 horses (17.65%) based on the ELISA test. Also, antibodies against *T. gondii* were detected in 41 horses (8.42%) based on IFAT and in 63 of 487 horses (12.94%) based on the ELISA test. Also, in 6 of the horses (1.23%) based on IFAT and in 13 of the 487 horses (2.67%) based on the ELISA test, double positivity suggested co-infection with both parasites. Gender, age groups, and the presence of dogs for neosporosis, and age groups and the presence of cats for toxoplasmosis, could be considered factors having an influence on the seroprevalences (P < 0.05). The results proved the importance of the urgent implementation of stringent regulatory measures to prevent and control the spread of these parasites.

## Introduction

*Neospora* spp. and *Toxoplasma gondii* are closely related parasites. They are globally distributed and belong to the apicomplexan group. These parasites share many morphological, structural, and immunological features. Their life-cycle consists of three distinct stages. Firstly, there are rapidly proliferating tachyzoites causing acute disease. Secondly, there are slowly dividing bradyzoites forming tissue cysts in chronically infected individuals. Thirdly, oocysts are formed in the intestine of definitive hosts, shed into the environment, and become orally infective sporozoites. Despite these similarities, the two species are biologically distinct^1–3^.

In the case of *N. caninum*, dogs play a key role as definitive hosts, shedding oocysts into the environment, in which infective sporozoites develop within a few days^4,5^. Oral infection with oocysts can take place by many warm-blooded species as intermediate hosts, including wildlife, farm animals, and also horses^6,7^. *Neosporosis* causes reproductive problems among its hosts, leads to economic losses, and can also cause serious neurological disorders, most notably in dogs^4^. IFAT is the gold standard test for indirect detection of *N. caninum* infection^1,8^. However, this test cannot distinguish between *N. caninum* and *N. hughesi*^9^. In an immunocompetent host, *T. gondii* infection typically does not cause clinical signs. However, in situations of transient immunosuppression, such as pregnancy, parasites can exploit the temporal downregulation of a Th1-biased immunity, invade placental, and fetal tissues and cause abortion^10^.

In contrast to *N. caninum*, *T. gondii* has a high zoonotic potential and is capable of infecting all warm-blooded animal species as well as humans^11^. Felines act as definitive hosts, shedding oocysts with their feces, which, similar to *Neospora*, are orally infective. In addition, ingestion of raw meat containing tissue cysts with bradyzoites that develop in the muscle or cerebral tissues of intermediate hosts, such horses, can also lead to infection^12^. *T. gondii* infection normally does not affect immunocompetent hosts, although psychological disorders have been reported to be associated with *T. gondii* infection^13^. However, in situations such as primary infection during pregnancy, the parasite can cause severe fetal malformations or even abortion. In addition, reactivated toxoplasmosis in chronically infected patients is known to represent a serious complication in immunocompromised individuals^14^. The first-line strategy to diagnose *T.gondii* infections in farm animals is similar to *Neospora*, serological testing for the presence of IgG^15^.

The previous review study in Iran demonstrated the range of *N.caninum* infection in horses varied between 20 and 42.2%^16^. Also, other studies that evaluated titers of *T. gondii* through horses from other parts of Iran varied a range of 11.5–48.5% ^17–20^.

However, to the best of our knowledge, there is no data has been made available so far on the seroprevalence of *T. gondii* and *Neospora* spp. in horses in Tehran, the capital of Iran, which is the most important place for breeding horses in the country. The aim of this study was to evaluate of *Neospora* spp. and *Toxoplasma gondii* situations among horses in Tehran and identify potential risk factors associated with these infections, which can be useful for further implementation. Besides, comparing the results of the ELISA test with IFAT as the gold standard test.

## Results and discussion

41 out of 487 horses (8.42%, 95% CI 6.16–10.68) were seropositive for *T. gondii* by IFAT. This number is in the range of the reported global seroprevalence of 11.29% and slightly lower than was found earlier in Asia (9.02%)^12^. Also, the number of 52 out of 487 (10.67%, 95% CI 8.23–13.22) horses being seropositive for *Neospora* spp. in Tehran, resulting in IFAT is in the range of what has been previously reported on a global scale (14.65%)^21^. However, it is clearly lower than the reported overall *N. caninum* seroprevalence of 52% in animals in Iran^22^, or the prevalence of 21.64% reported for Asia^21^. Besides, double infections were noted in 1.23% of the horses investigated in this study (95% CI 0.44–2.03, 6/487) by IFAT (Table 1).

**Table 1:** Seroprevalence of *Neospora* spp. and *Toxoplasma gondii* infections in horses in Tehran, Iran.

Type of TEST	*Neospora* positive (%)	*Neospora* negative (%)	Total (%)
ELISA			
* Toxoplasma* positive (%)	13 (2.67%)	50 (10.27%)	63 (12.94%)
* Toxoplasma* negative (%)	73 (14.98%)	351 (72.08%)	424 (87.06%)
Total (%)	86 (17.65%)	401 (82.34%)	487 (100%)
IFAT			
* Toxoplasma* positive (%)	6(1.23%)	35(7.18%)	41(8.41%)
* Toxoplasma* negative (%)	46(9.44%)	400(82.13%)	446(91.58%)
Total (%)	52(10.67%)	435(89.32%)	487 (100%)

When looking at the animal characteristics, the age of the animals could be regarded as a factor that affects the seroprevalence in horses for both parasites. Adult horses over 24 months of age exhibited a significantly higher *Neospora* seroprevalence (25.84%) compared to foals (8.49%) and yearlings (11.03%) (P < 0.05) by analyzing ELISA results. Moreover, 17.79% of adult horses had a significantly higher *T. gondii* seroprevalence compared to foals (6.6%) and yearlings (17.79%) (P < 0.05) (Tables 2, 3). This result is not surprising as it reflects the timing of the potential postnatal exposure of horses to oocysts, which is regarded as the main transmission route of herbivores^1,21,23^. As a result, with increasing age, the chance of being exposed to infected water and food increases, thus it is expected to have a higher prevalence among adults. Another factor that has a significant relationship with prevalence is the presence of the respective definitive hosts in the vicinity of the horse stables. The presence of dogs was associated with seropositivity for *N. caninum,* and the presence of cats was significantly associated with *T. gondii* infection (21.05% and 16.23% by ELISA, respectively, P < 0.05) (Tables 2, 3). However, other studies have demonstrated there is no positive correlation between the persistence of definitive hosts and *N. caninum* or *T. gondii* seroprevalence^14,24^.

**Table 2:** Seroprevalence rate of *Neospora* infections in horses from Tehran in different variables.

Characteristics	Horses tested	*Neospora* spp.		
		IFAT positive (%)	cELISA positive (%)	*p-value*
Gender				
Female	261	15 (5.74%)	29 (11.11%)	cELISA (*P* = 0.000*) IFAT (*P* = 0.000*)
Male	226	37 (16.37%)	57 (25.22%)	
Age categories (years)				
Foals (< 12 months)	106	7 (6.6%)	9 (8.49%)	cELISA (*P* = 0.000*) IFAT (*P* = 0.000*)
Yearlings (12–24 months)	145	12 (8.27%)	16 (11.03%)	
Adults (> 24 months)	236	33 (13.98%)	61 (25.84%)	
Breed				
Arab	98	9 (12.24%)	16 (16.32%)	cELISA (*P* = 0.579) IFAT (*P* = 0.267)
Turkmen	85	7 (11.76%)	13 (15.29%)	
Dareshuri	76	6 (6.57%)	11 (14.47%)	
Mix	228	30 (10.96%)	46 (20.17%)	
Keeping status				
Entertainment	296	33 (11.14%)	57 (19.25%)	cELISA (*P* = 0.475) IFAT (*P* = 0.776)
Sport	105	11 (10.47%)	17 (16.19%)	
Breeding	86	8 (9.3%)	12 (13.95%)	
Type of breeding				
Natural breeding	325	37 (11.38%)	59 (18.15%)	cELISA (*P* = 0.685) IFAT (*P* = 0.495)
Artificial insemination	162	15 (9.25%)	27 (16.66%)	
Presence of dogs				
Yes	304	39 (12.82%)	64 (21.05%)	cELISA (*P* = 0.011*) IFAT (*P* = 0.047*)
No	183	13 (7.1%)	22 (12.02%)	

*Statistically significant (P-value ≤ 0.05).

**Table 3:** Seroprevalence rate of *Toxoplasma* infections in horses from Tehran in different variables.

Characteristics	Horses tested	*Toxoplasma gondii*		
		IFAT positive (%)	cELISA positive (%)	*p-value*
Gender				
Female	261	18 (6.89%)	29 (11.11%)	cELISA (*P* = 0.197) IFAT (*P* = 0.193)
Male	226	23 (10.17%)	34 (15.04%)	
Age categories (years)				
Foals (< 12 months)	106	6 (5.66%)	7 (6.6%)	cELISA (*P* = 0.006*) IFAT (*P* = 0.032*)
Yearlings (12–24 months)	145	11 (7.58%)	14 (9.6%)	
Adults (> 24 months)	236	24 (10.16%)	42 (17.79%)	
Breed				
Arab	98	8 (8.16%)	13 (13.26%)	cELISA (*P* = 0.676) IFAT (*P* = 0.588)
Turkmen	85	6 (7.05%)	10 (11.76%)	
Dareshuri	76	5 (6.57%)	7 (9.21%)	
Mix	228	22 (9.64%)	33 (14.47%)	
Keeping status				
Entertainment	296	27 (9.12%)	40 (13.51%)	cELISA (*P* = 0.884) IFAT (*P* = 0.527)
Sport	105	8 (7.61%)	13 (12.38%)	
Breeding	86	6 (6.97%)	10 (11.62%)	
Type of breeding				
Natural breeding	325	29 (8.92%)	43 (13.23%)	cELISA (*P* = 0.784) IFAT (*P* = 0.696)
Artificial insemination	162	12 (7.40%)	20 (12.34%)	
Presence of cats				
Yes	234	28 (11.96%)	38 (16.23%)	cELISA (*P* = 0.037*) IFAT (*P* = 0.006*)
No	253	13 (5.13%)	25 (9.88%)	
Presence of mice				
Yes	182	19 (10.43%)	29 (15.93%)	cELISA (*P* = 0.128) IFAT (*P* = 0.216)
No	305	22 (7.21%)	34 (11.14%)	

*Statistically significant (P-value ≤ 0.05).

There were clear differences between genders. *Neospora* spp. antibodies were more prevalent in males (25.22%) compared to female horses, indicating that male horses could be more prone to infection (Table 2). However, more studies need to be carried out to confirm there are no other influencing variables, such as environmental contamination. In this respect, earlier studies have reported that there is no association between *Neospora* seroprevalence and sex in horses^25,26^.

For females, the impact on how breeding (e.g., natural, artificial, and insemination) would influence them had no significant impact on seroprevalence values. However, the numbers were slightly higher in the case of natural breeding (18.5% for *Neospora*, and 13.23% for *Toxoplasma* by ELISA) (Tables 2, 3).

In addition, there were also no significant differences between breeds, and *T. gondii* also *Neospora* spp. seroprevalence. A slightly higher prevalence was recorded in mixed breeds for *Neospora* and *T. gondii* (Tables 2, 3). Similar findings have been reported earlier in a study carried out in Western Para, Brazil^27^. The most likely explanation is that the management of pure-breed horses is more elaborate and controlled than for mixed breeds, and this would have an impact on the infection pressure^27^. In terms of the use of these horses for entertainment, sport, or breeding, no seroprevalence differences could be found, indicating that the keeping status did not have an impact. However, seroprevalences in entertainment horses were increased for both parasites, most likely due to the greater chance of facing definitive hosts and oocysts^27^ (Tables 2, 3).

IFAT and ELISA are widely used serological tests for the detection of *N. caninum* and *T. gondii* infections. Both tests have high sensitivity and specificity, allowing for accurate detection of *N. caninum* and *T. gondii* infections. Besides, previous researchers used IFAT or ELISA, and in some cases, both tests, to detect those pathogens^26,28–30^.

**Figure 1:**
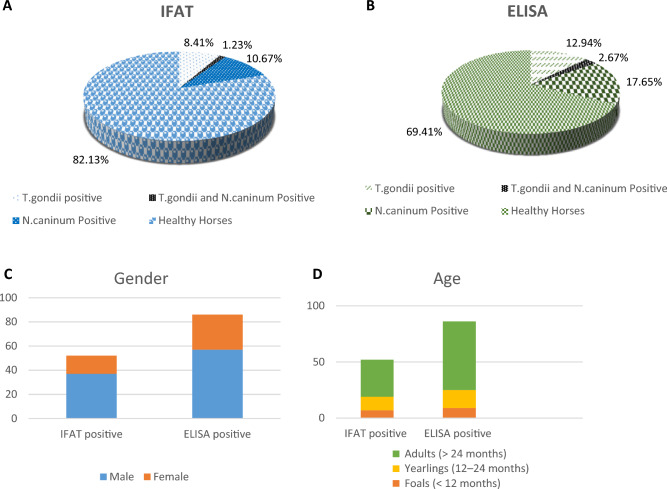
The comparative results of IFAT and ELISA for the seroprevalence of both pathogens are shown in (**A**, **B**), respectively. Additionally, the comparative results of IFAT and ELISA for both pathogens are depicted in the results of significant risk factors in (**C**, **D**).

Moreover, our results have shown ELISA could be a good detection technique for both parasites in cases of a lack of a gold standard test and time and budget limitations (Fig. 1).

Unfortunately, there is no information on whether these animals have any history of abortion or other disease patterns, which could be correlated with *neosporosis* or *toxoplasmosis*. There is seroprevalence data available from other cities in Iran^12,21,22^, but the small number of exposed animals renders the data less reliable.

## Conclusion

This study is the first report on *T. gondii* and *N. caninum* seropositive horses in Tehran. According to the results of the seroprevalence of *N. caninum* (10.67%) and *T. gindii* (8.41%) in horses from Tehran, one of the messages from this study is that horse meat originating from Tehran should be consumed with caution, and consumption of raw milk from seropositive horses could be a potential source of toxoplasmosis. Another message is to implement robust regulatory measures to prevent and manage the spread effectively.

## Materials and methods

### Study design

**Figure 2:**
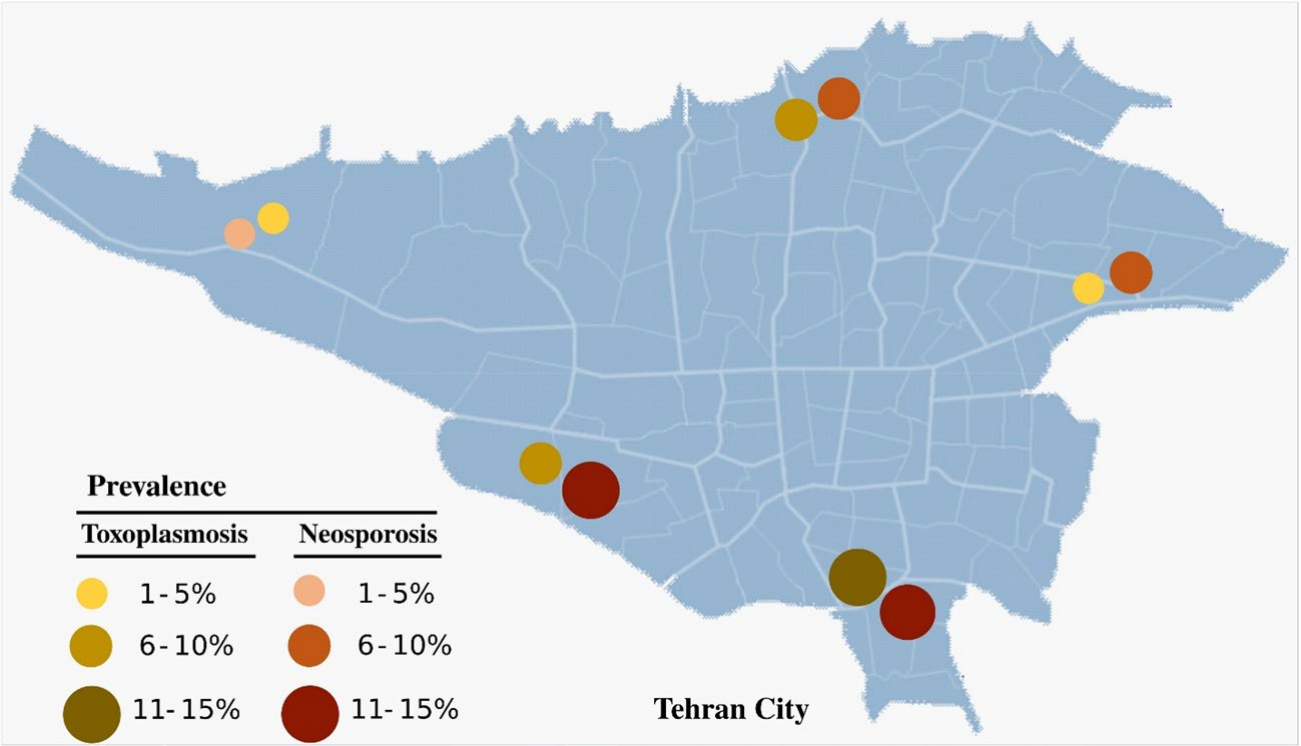
The graduated symbol map of the prevalence of neosporosis and toxoplasmosis in different regions of Tehran City, Iran.

The present study is a cross-sectional study that was conducted to investigate the seroprevalence of *Neospora* spp. and *Toxoplasma gondii* in horses from the capital city of Iran, Tehran. A web-based software, EpiTools (Epidemiological Calculators; https://epitools.ausvet.com.au/), was used to calculate the sample size based on the following assumptions: (1) in consideration of the limited information available about *Neospora* spp. and *Toxoplasma gondii* among horses in Tehran, we assumed a prevalence of 50%, (2) a confidence level of 95%, and (3) the precision of 5% for the unknown population size in the estimation^4^. Tehran has 69 horse stables. The researchers selected 487 clinically healthy horses from 17 stables during September and November 2022 by respecting the different geographical regions, the demographics of the horse population (such as age, breed, and sex), the management practices employed at each facility (mechanized stables), the accessibility of the stables for sampling, and a good history of animal health management and veterinary care to minimize confounding variables. Additionally, obtaining consent and cooperation from stable owners or managers was crucial for successful data collection. There was no significant difference in the prevalence of neosporosis and toxoplasmosis between different areas of Tehran city (P < 0.05) (Fig. 2). Blood samples were collected from the jugular veins of horses in sterile vacuum tubes without anticoagulants; after that, serum was separated by centrifugation and stored at − 20 °C until further analysis. Furthermore, to identify samples from horse-populated areas, a cluster sampling technique was employed^30^. During sample collection, owners provided information on particular animal characteristics (gender, age, and breed), as well as on local management and environmental conditions (presence of cats, mice, and dogs, equine use, and breeding methods). All this information was recorded for the subsequent statistical evaluation of any significant associations with seroprevalence.

### Serology

To identify antibodies against *Toxoplasma gondii* and *Neospora* spp., we employed the indirect fluorescence antibody test (IFAT). We used commercially available IFR antigens for both pathogens (VMRD, Pullman, Chicago, USA) and an anti-horse IgG FITC conjugate (VMRD). Serum samples underwent a stepwise two-fold dilution, starting at 1:50, with a positive result defined as a titer of 50 for both tests. The process involved fixing the antigens on glass slides, adding serum, and incubating in a humid chamber at 37 °C. After washing, drying, and applying the specific conjugate, the slides were incubated for an additional 30 min and examined under a fluorescence microscope (OLYMPUS BX 41) at 1000 × magnification with oil immersion, looking for continuous peripheral fluorescence to confirm specificity. We used horse sera screened by latex agglutination and IFAT as positive and negative controls for both pathogens^29^.

For anti-* Neospora* spp. IgG detection, we used a competitive ELISA (cELISA, VMRD, Inc., Pullman, WA) according to the manufacturer's instructions. Samples with a percent inhibition of ≥ 30% were considered *Neospora* positive. To detect *T. gondii* IgG, we used a commercial Toxoplasmosis ELISA kit (IDVET, Montpellier, France) following the manufacturer's instructions. Seroprevalence was determined by measuring OD at 450 nm and calculating the positive percentage (S/P). Results were categorized as negative (< 40%), doubtful (40–50%), or positive (> 50%).

### Data analysis

Seroprevalence and statistical associations were analyzed using the statistical software package R 4.2.1^31^. In other words, the epiR package was used to estimate seroprevalence and its confidence intervals^32^. Moreover, Pearson’s chi-square test was utilized to evaluate the association between serological status and epidemiological factors, and (P < 0.05) was set as the level of significance.

### Ethics approval

The study protocol was approved by the Iran national Ethics Committee on the Iran Islamic Azad University, Karaj, Research Ethics Committee IR.IAU.K.REC1401.06. All experiments were performed according to the relevant guidelines and regulations. The study was carried out in compliance with the ARRIVE guidelines.

### Consent to participate

All the authors consent to participate in publication.

## Data Availability

Data are available upon request from the corresponding author.
